# Patient-reported assessment of compassion in Spanish: a systematic review

**DOI:** 10.3389/fmed.2024.1352694

**Published:** 2024-07-10

**Authors:** Ana Soto-Rubio, Carmen Picazo, Beatriz Gil-Juliá, Yolanda Andreu-Vaillo, Marian Pérez-Marín, Shane Sinclair

**Affiliations:** ^1^Developmental and Education Psychology Department, Faculty of Psychology and Speech Therapy, University of Valencia, Valencia, Spain; ^2^Psychology and Sociology Department, University of Zaragoza, Zaragoza, Spain; ^3^Personality, Assessment, and Psychological Treatments Department, Faculty of Psychology and Speech Therapy, University of Valencia, Valencia, Spain; ^4^Compassion Research Lab, Faculty of Nursing, University of Calgary, Calgary, AB, Canada; ^5^Division of Palliative Medicine, Department of Oncology, Cumming School of Medicine, University of Calgary, Calgary, AB, Canada

**Keywords:** compassion, assessment, compassionate care, health care, systematic review, measure, patient reported outcome measure (PROM), Spanish

## Abstract

**Aims and objectives:**

This systematic review aims to: (1) explore which tools have been used in Spanish to measure compassion; (2) know which of these tools could be used to assess compassion in healthcare settings from the perspective of patients; (3) evaluate the quality of these patient-reported measures in Spanish contexts; and (4) determine which of these instruments would be best suited to be used in healthcare settings.

**Background:**

Compassion has been recognized as a fundamental dimension of quality healthcare.

**Methods:**

Several scientific databases were consulted for relevant records published up to December 16^th^, 2021. In accordance with PRISMA guidelines, 64 studies were included.

**Results and conclusions:**

while existing instruments, validated in Spanish, allow for the measurement of self-compassion or compassion to others, there are no valid and reliable measures currently available in Spanish to measure patient-reported compassion.

**Relevance to clinical practice:**

In order to ensure and promote compassion in the health care context, it is essential to have a valid and reliable tool to measure this construct in a patient-informed way, and this is currently not possible in the Spanish-speaking context because of the lack of such an instrument in Spanish.

## Introduction

In healthcare, the importance of providing patient-informed care is increasingly recognized. This explains the increasing use of Patient Reported Outcomes (PROs) as key means for including the patient’s perspective in the assessment of quality care. Among the different PROs, Patient Reported Experience Measures (PREMs) are those patient-reported measures that directly assess the patient’s experience of some aspect of care (1, 2).

Concurrently, compassion has been recently identified as an essential indicator of quality care, requiring researchers to develop an understanding of how patients perceive and experience compassion in healthcare (3–5). Thus, a patient-informed, empirical definition of compassion was recently developed: “a virtuous response that seeks to address the suffering and needs of a person through relational understanding and action” (6). In this sense, compassion is not restricted exclusively to physical aspects related to pain and symptom management but considers all of the patient’s holistic needs. In recognition of the centrality of compassion to patients’ experience of quality care, compassion has increasingly been taken into consideration in international clinical practice standards and policies, which recognize compassion as a fundamental dimension of quality care that needs to be integrated into the practice and education of healthcare providers and embedded within the larger healthcare system (5, 7–9).

Notably, the absence of compassion in health care has been linked to poor symptom control, patient complaints, high chronic stress, and medical complications (3, 4, 9–12). At the same time, the presence of compassion in healthcare has been linked to greater emotional well-being in patients, improved quality of life, and greater satisfaction with care (4, 5, 9, 13–16). Furthermore, several studies on quality care in healthcare indicate that patients identify compassion as one of their most important unmet needs (3, 5, 9, 13, 14).

The significant impact of compassion on various indicators of healthcare quality has led researchers, educators, and policymakers worldwide to consider compassion as: (1) a patient right (17), (2) a core professional competency (18–20), and (3) a standard of care that healthcare organizations, educators, and care providers need to implement, measure, evaluate, and report on (5, 7, 16, 21–23). Despite the recognition of the importance of compassion in research, education, policy, and practice, there is a gap between what is known in theory and integration into practice (3–5, 16, 24–26).

Several explanatory factors play a role in this paradoxical situation between the direction healthcare practice should be heading and the day-to-day reality of practice. Indeed, the difficulty of defining compassion and distinguishing it from other related terms such as empathy or sympathy; the different connotations that the term has in different languages or cultures; the difficulty of operationalizing the concept due to its subjective nature are among the possible reasons for such a discrepancy (27, 28). Closely related to all of them, the absence of valid and reliable patient-reported measurement tools (PREMs) to assess patient care and the impact of training and intervention programs aimed at fostering compassion in the healthcare setting arises as a key obstacle.

The relevance of the use of PROs has been widely recognized in the scientific literature and by health authorities, including those from Spanish-speaking countries or countries with a significant Spanish-speaking population (29). Thus, it is pivotal to know which patient-reported instruments have been used to assess compassion and their psychometric properties in order to ensure that this standard of care and this reputed patient right is assessed in a psychometrically rigorous manner.

Therefore, the aims of this systematic review are: (1) to explore which compassion measures have been used in Spanish settings; (2) to determine which tools are capable of assessing compassion in healthcare from the patient’s perspective; (3) to evaluate the quality of these patient-reported compassion measures in Spanish contexts; and (4) to assess which of these instruments would be best suited to be used in Spanish-speaking patient populations.

## Methods

This systematic review was conducted according to the Preferred Reporting Items for Systematic Reviews and Meta-Analyses (PRISMA) standard (30).

### Inclusion and exclusion criteria

Studies that met the following criteria were included in this systematic review: (a) the study included the assessment of compassion in Spanish, (b) the study was published in peer-reviewed journals, and (c) the study was published in English or Spanish.

As exclusion criteria, the following were considered: (a) assessments of other subsidiary constructs (such as “compassion fatigue” or “compassion satisfaction”), (b) papers published in conferences, (c) narrative reviews, and (d) single-case designs.

### Bibliography search

The ProQuest Central, PubMed, Web of Science, Embase, and ÍnDICEs databases were consulted by two authors for relevant records published up to 16th December 2021. In line with the FINER (Feasible, Interesting, Novel, Ethical, and Relevant) criteria (31) the following research question guided this review: Which instruments have been used to assess compassion in Spanish? The final search combined the proposed keywords: Spanish, Compass* (compassion, compassionate), and measure* (measure, measurement, measuring) or assess* (assess, assessment, assessing).

The following Boolean expression was therefore used in Pro-Quest, Web of Science, PubMed, and ÍnDICEs: ((Spain OR Spanish) AND (compass*) AND (measur* OR assess*)), and in Embase, the following: ((“Spain”/exp. OR “Spanish”/exp) AND compass* AND (measur* OR assess*)). The fields selected were title and/or abstract, and the languages specified were English or Spanish.

All the retrieved articles were uploaded to Covidence (*Covidence systematic review software*, 2021), an online screening and data extraction tool. After removing duplicate articles, two authors reviewed all the papers’ titles and abstracts and excluded those articles that did not meet the inclusion criteria. When discrepancies emerged between the reviewers, articles were re-read in-depth independently, re-evaluated, and discussed to reach a consensus. A third researcher (SS) was available to resolve discrepancies if necessary, which was not required as all discrepancies were resolved by the initial two reviewers. To evaluate the interrater agreement index Cohen’s Kappa (κ) was used (32) following the guidelines suggested by Landis and Koch (33), where less or equal to 0.39 is considered poor agreement, between 0.40 and 0.75 is considered moderate, and between 0.75 and 1 is considered excellent (33).

### Extraction of relevant data: full-text review

One author developed a data extraction form to obtain relevant information from the included studies. This information included general information such as: Title of paper, Authors, Year of publication, Country in which the study was conducted, Characteristics of the study (Methods, Aim of study, Study design, Participants, Population description, Inclusion criteria, Exclusion criteria, Total number of participants) and specific information about measure of compassion (Name of instrument, Authors, Year, Type of compassion, Spanish validation (where, by whom, when), total items, scales, psychometric properties). All authors considered this data extraction form to be appropriated. Data extraction was carried out independently by two authors and consensus was reached with a third party when there where discrepancies.

## Results

### Study selection and screening

**Figure 1:**
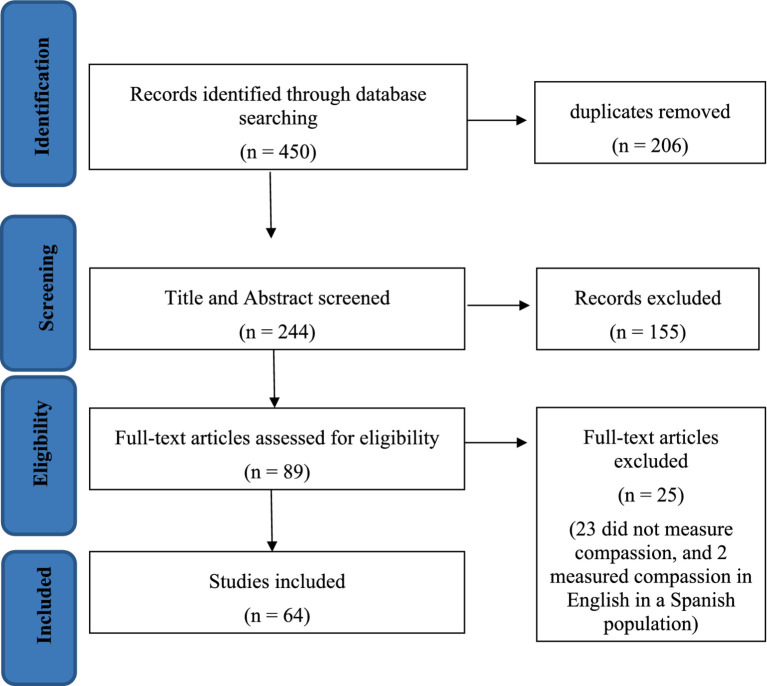
PRISMA flow diagram for the systematic review.

Our search resulted in a total of 450 records being identified across five databases (Figure 1). After removing duplicates, the total number of records was reduced to 244. Of the 244 articles that underwent title and abstract screening, 155 studies were excluded, resulting in 89 articles being reviewed in full. At this stage, 25 additional manuscripts were excluded, resulting in 64 studies being included in this review. The inter-rater reliability index (Cohen’s Kappa) between the two independent reviewers in the full-text screening was excellent (κ = 0.80).

### Characteristics of the studies that measure compassion in Spanish

**Figure 2:**
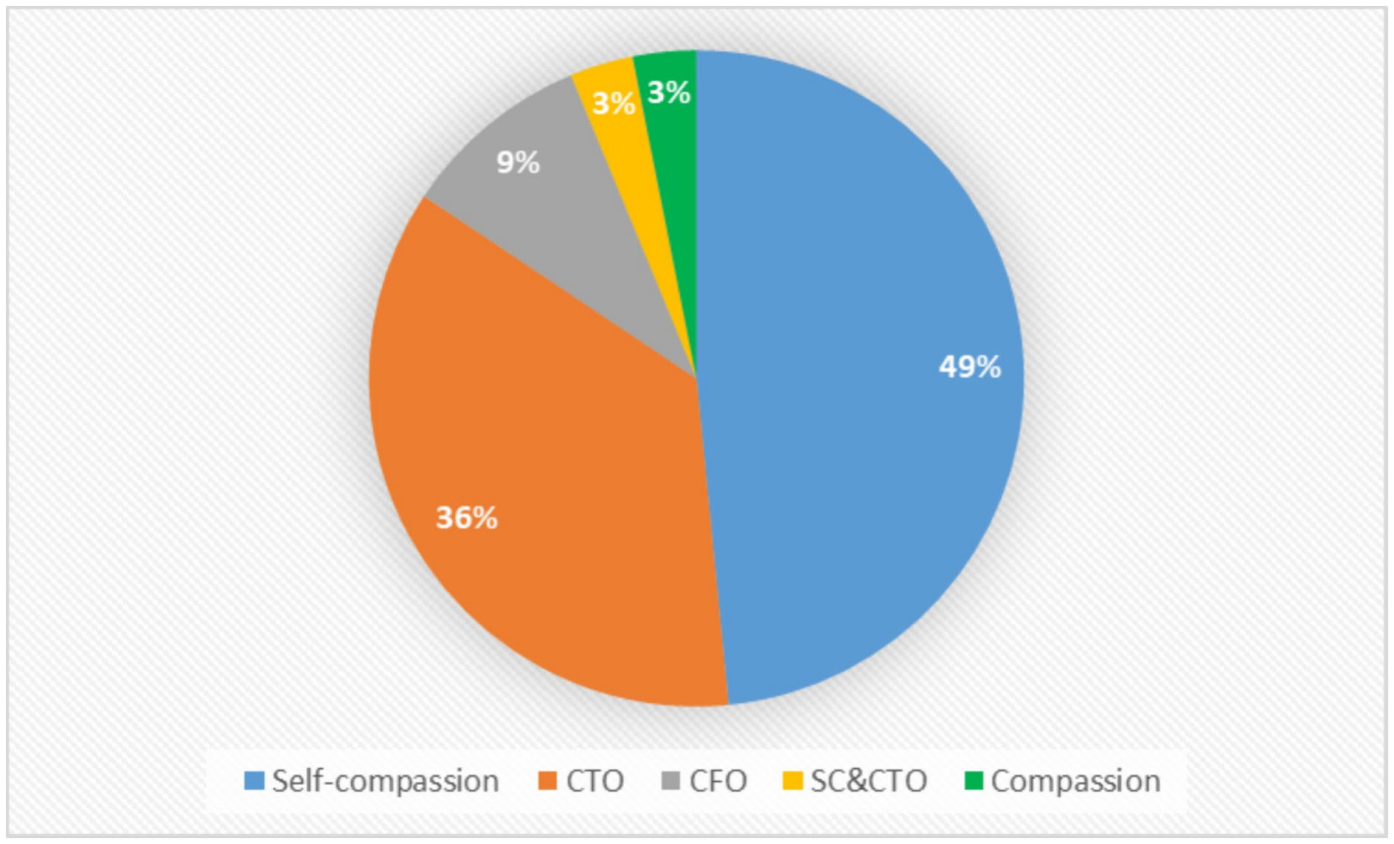
Studies assessing different types of compassion in Spanish. CTO, Compassion Toward Others; CFO, Compassion From Others; SC&CTO, Self-compassion and Compassion Toward Others.

From the 64 studies that measured compassion in Spanish, 31 (47.7%) measured self-compassion, 23 (36.9%) compassion toward others, 6 (9.2%) compassion from others, 2 (3.1%) simultaneously assessed self-compassion and compassion toward others, and 2 (3.1%) compassion in general (See Figure 2). Thirty-five studies (53.8%) included the psychometric properties of the instruments used in a Spanish sample. Fifty-three studies (81.5%) used a validated Spanish version of the instrument assessing compassion. Table 1 shows the distribution of the studies by type of compassion being assessed, year of publication, country of the study, inclusion of a Spanish-validated version of the instrument used, and inclusion of the instrument’s psychometric properties for the study sample. The characteristics of the instruments used to measure compassion in Spanish are summarized in Table 2.

**Table 1:** Characteristics of the studies included.

Form of compassion (*n*)	Year (*n*)	Country (*n*)	Spanish val. (*n*)	Psychometrics (*n*)
Self-compassion (31)	2007 (1)	Spain (25)	Yes (31)	Yes (16)
	2013 (1)	United States (2)	No (0)	No (15)
	2014 (1)	Latin America (1)		
	2016 (4)	Chile (1)		
	2018 (4)	Argentina (2)		
	2019 (5)			
	2020 (5)			
	2021 (10)			
Compassion toward others (24)	2003 (1)	Spain (13)	Yes (17)	Yes (16)
	2005 (1)	Mexico (3)	No (7)	No (8)
	2006 (1)	Argentina (2)		
	2007 (1)	Colombia (1)		
	2009 (3)	Dominican Republic (1)		
	2011 (2)	Ecuador (1)		
	2014 (1)	Peru (1)		
	2016 (2)	Chile (1)		
	2018 (3)	Latin America (1)		
	2020 (5)	Several (1)		
	2021 (4)			
Compassion from others (6)	2009 (1)	United States (3)	Yes (3)	Yes (1)
	2014 (1)	Colombia (2)	No (3)	No (5)
	2015 (1)	Spain (1)		
	2017 (1)			
	2018 (1)			
	2019 (1)			
Self-compassion and Compassion toward others (2)	2013 (1)	Spain (2)		Yes (1)
	2021 (1)		No (2)	No (1)
Compassion (2)	2021 (2)	Spain (2)	Yes (2)	Yes (1)
				No (1)
Total (64)	2003 (1)	Spain (42)	Yes (53)	Yes (35)
	2005 (1)	USA (5)	No (11)	No (29)
	2006 (1)	Argentina (4)		
	2007 (2)	Mexico (2)		
	2009 (4)	Colombia (3)		
	2011 (2)	Chile (2)		
	2013 (2)	Latin America (2)		
	2014 (2)	Dominican Republic (1)		
	2015 (1)	Ecuador (1)		
	2016 (6)	Peru (1)		
	2017 (1)	Several (1)		
	2018 (8)			
	2019 (6)			
	2020 (10)			
	2021 (17)			

**Table 2:** Characteristics of the instruments used to assess in Spanish a form of compassion.

Form of compassion	Studies (*n*)	Instrument	Original Authors	Year original	Versions	Total items	Items compassion	Scales	Authors validation	Year validation	Population of validation	Psychometrics
Self-compassion (31)	(34–64) (31)	SCS Self-Compassion Scale	Neff (65)	2003	Long	26	26	Six: Self-kindness, self-judgment, common humanity, isolation, mindfulness, over-identification.	García-Campayo et al. (34)	2014	Spanish population	
					Short	12	12	Six: Self-kindness, self-judgment, common humanity, isolation, mindfulness, over-identification.	García-Campayo et al. (34)	2014	Spanish population	
Compassion toward others (23)	(66–73) (8)	JSPE Jefferson Scale of Physicians (or Nursing) Empathy	Hojat et al. (74)	2001	Short	20	2	Three: taking perspective, compassionate attention, and ability to put oneself in the patient’s shoes.	Blanco et al. (67)	2018	Spanish medical students	Cronbach Alpha 0.8–0.9
									Alcorta et al. (66)	2005	Mexican medical students	
	(41, 47, 75) (3)	CS Compassion Scale	Pommier et al. (76)	2010		16	16	Four: Greater kindness, common humanity, mindfulness, and lessened indifference. Sousa et al. (2017) propose two higher-order factors: compassion and disconnectedness.	Not validated			
	(77) (1)	TCI-R Temperament and Character Inventory—Revisited	Cloninger et al. (78)	1994				Seven dimensions with traits. Compassion is one of the five traits of the cooperativeness dimension (The ability to cooperate and identify with other people). Compassion vs. revengefulness continuum.	Apud et al. (77)	2020	Former substance users from Catalonia and surrounding areas	Moderate/high internal consistency
	(79) (1)	SCBCS Santa Clara Compassion Scale	Hwang et al. (80)	2008		5	5		Caycho-Rodríguez et al. (79)	2020	Peruvian university students	Cronbach Alpha = 0.9
	(81) (1)	Dictator, Ultimatum, and Trust Games	Exadaktylos et al. (124)	2013		2	2	Compassion is defined as “how much one suffers from advantageous inequality.” Alpha (envy): “I am not worried about how much money I have; what worries me is that there are people that have more money than I have” Beta (compassion): “I am not worried about how much money I have, what worries me is that there are people who have less money than I have.”	Not validated			
	(82) (1)	OMS Opening Minds Scale	Kassam et al. (83)	2012		20	1	Compassion toward patients	Gajardo et al. (82)	2021	Healthcare practitioners in Chile	
	(84) (1)	CCS Caregiving Compassion Scale	Schulz et al. (85)	2017		10	10	Two factors: (1) Distress from witnessing the care recipient suffering and (2) Motivation and disposition to helping	Gallego et al. (84)	2021	Spanish family caregivers of people with dementia		
(86) (1)	COOL Compassion for Others’ Lifes	Chang et al. (86, 87)	2014		26	26	Two subscales: empathy (13 items) and alleviating suffering (13 items)	Klos et al. (88)	2020	Latin-Americans		
(89) (1)	APS Affective Picture System	Lang et al. (90)	2005		28 images	28	Four scales: Valence, Arousal, Dominance and Compassion	Mercadillo et al. (89)	2007	Mexicans		
(91) (1)	IRI Interpersonal Reactivity Index	Davis (92)	1980		28	7	Four scales, seven items each: Perspective-taking, Fantasy, Empathic concern (which measures feelings of warmth, compassion, and concern for others), and personal distress					
(93) (1)	Transcendent values	Schwartz (94)	2007		5	5	Self-transcendent values	Pizarro et al. (93)	2021	Spanish university students	Cronbach Alpha = 0.751	
(95) (1)	DSES Daily Spiritual Experience Scale	Underwood (96)	2006		16	2	Compassionate love (describes moments when people stretch out to those around them in care and acceptance). “I feel a selfless caring for others,” and “I accept others even when they do things I think are wrong.”	Mayoral et al. (97)	2013	Mexicans		
(98) (1)	VASCUETHICS Questionnaire	Clará et al. (98)	2006		5	4	Five clinical ethic dilemmas, of which 4 presented a conflict between compassion toward a “small” or “very costly” beneficial action vs. a reasonable but more “pragmatic” allocation of health resources	Clará et al. (98)	2006	Spanish vascular surgeons		
(99) (1)	CLSH Compassionate Love Scale for Humanity	Chiesi et al. (100)	2020	Short	9	9	Compassion or altruistic love toward strangers, selfless caring, and motivation to help humanity	Miragall et al. (99)	2021	Spanish population	Cronbach Alpha = 0.91	
		Sprecher et al. (101)	2005	Long							
Compassion from others (6)	(102) (1)	DHC Dental Home Concept	Rozier et al. (102)	2019		10	3	Scales: accessible-comprehensive, compassionate, and health-literate care	Rozier et al. (102)			
	(103, 104) (2)	PCRS Professional Care Rating Scale	Swanson (105)	1993		15	7	Compassionate healer (7 items) and competent healer (8 items)	Posada-Morales (106)	2011		
									Vesga-Gualdrón (107)	2013	Pregnant women in Colombia	Cronbach Alpha = 0.893	
(108) (1)	IPC Interpersonal Processes of Care	Steward et al. (107)	2007	Short			Three communication scales: lack of clarity, elicited concerns/responses, and explained results. One patient-centered decision-making scale: decided together. Three interpersonal styles scales: compassionate/respectful, discriminated due to race/ethnicity, and disrespectful office staff.	Not validated				
(108) (1)	Narrative elicited by interview, family-centered care model	Moore et al. (108)	2015				Three major themes emerged: compassionate communication; capacity building for families, providers, and facilities; and coordination of care transitions.	Not validated				
(109) (1)	PROMIS Patient-Reported Out-Comes Management System	Gregory et al. (109)	2013			1		Not validated			
Self-compassion and Compassion Toward Others (2)	(110) (1)	SOFI Self-Other Four Immeasurables	Kraus et al. (111)	2009		16	16	Positive qualities toward self, positive qualities toward others, negative qualities toward self, negative qualities toward others	Not validated			
	(112) (1)	Narratives elicited by paintings	Karkabi et al. (112)	2013		Three paintings			Not validated			
Compassion (2)	(48) (1)	OSDS Osgood Semantic Differential Scale	Osgood (113)	1964		23		Evaluative (8), potency (8), activity (7)	Not validated			
	(114) (1)	RCS Relational Compassion Scale	Hacker (115)	2008		16	16	Self-self (4), self-others(4), others-self (4), others-others (4)	García-Campayo et al. (34)	2014		

**Figure 3:**
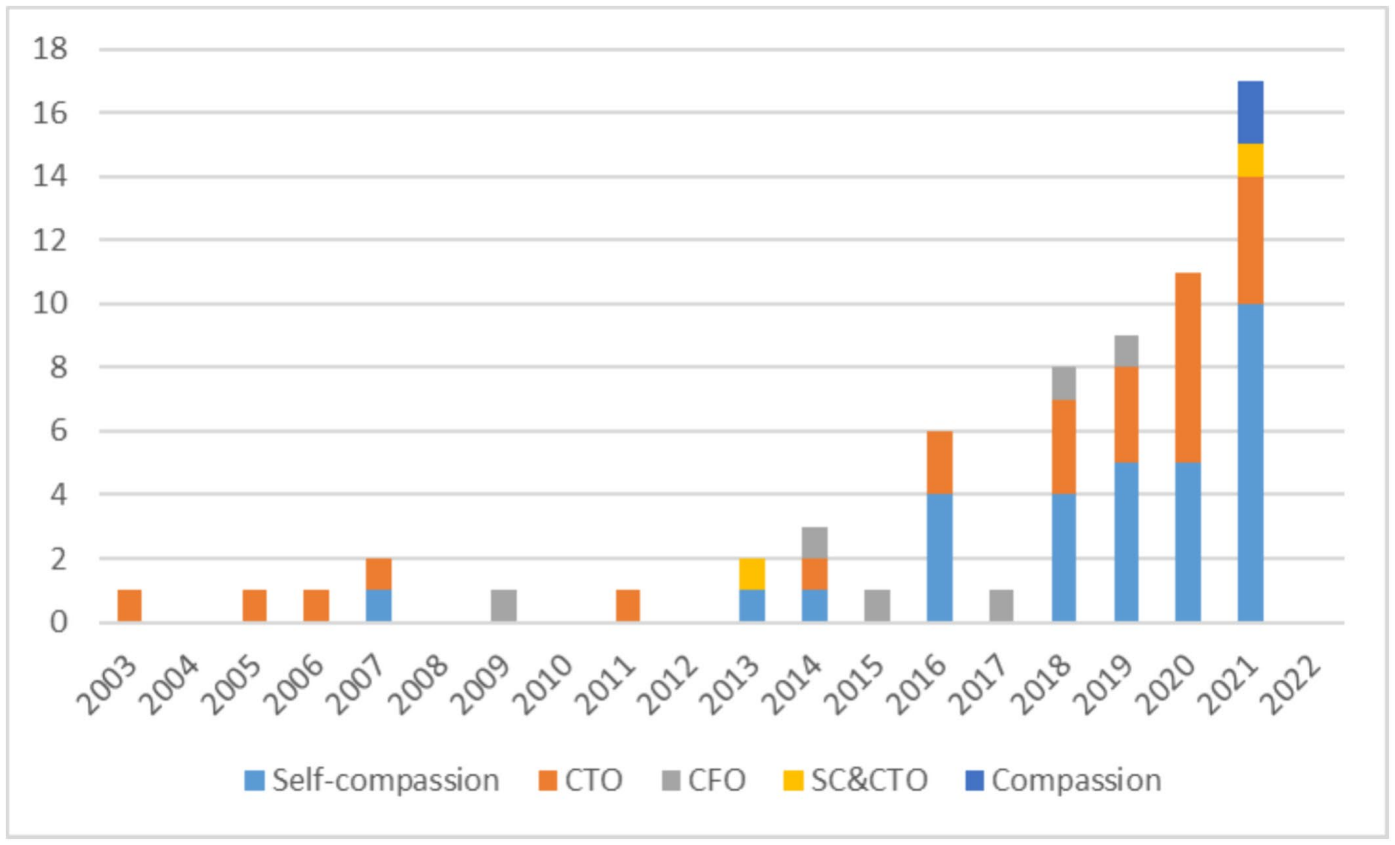
Number of publications assessing compassion in the language of Spanish. CTO, Compassion Toward Others; CFO, Compassion From Others; SC&CTO, Self-compassion and Compassion Toward Others.

The results show that there has been a rapid influx of compassion research across Spanish populations. However, no systematic reviews to date have synthesized and evaluated the evidence associated with this burgeoning field of scholarship. An overview of the number of publications where compassion has been assessed in Spanish per year can be found in Figure 3.

### Evaluation of instruments assessing self-compassion, compassion to others, and general compassion

Regarding the measures utilized in studies assessing self-compassion in a Spanish context, all of them exclusively used a translated version of the Self-Compassion Scale (65), validated in Spanish by García-Campayo et al. (34).

Regarding measures used to assess compassion toward others in Spanish populations, the instruments used were more varied than in the case of the assessment of self-compassion. However, by far the most frequent tool used was the Jefferson Scale of Physicians Empathy (JSPE) (74), which has been validated in Spanish in two separate populations: one in Mexican medical students (66) and one in Spanish medical students (67). From the tools used to assess compassion toward others, besides the JSPE, the Compassion Scale (CS) (76) was the only one that was used in more than one study; however, it has not been validated in Spanish. All the instruments used to assess compassion toward others are shown in Table 2.

The two studies that measured compassion with instruments that aimed to simultaneously assess compassion felt toward others (compassion for others) and toward oneself (self-compassion), one used a projective technique and a qualitative approach (112). The other used the Self-Other Four Immeasurables (SOFI) tool by Kraus and col. (111), which focuses on positive/negative feelings toward oneself/others and not on the construct of compassion specifically, and neither of these two assessment tools has been validated in Spanish.

Furthermore, two studies measured compassion from multiple perspectives (assessing compassion from others, toward others, between others, and toward oneself). One study used the Osgood Semantic Differential Scale (113), a *semantic* rating *scale* measuring the connotative meaning of multiple concepts, including compassion. The second study measured compassion using the Relational Compassion Scale (RCS) (115). The RCS assesses compassion via four subscales, with four items each: self-self, self-others, others-self, and others-others.

Of the six studies that measured compassion as received from others (i.e., from the recipient’s point of view), only three used a measurement tool that had been validated in a Spanish setting, with no studies utilizing a patient-reported experience measure. All three studies were conducted in the United States, and none were among patient populations. Moreover, just one provided an indicator of the instrument’s psychometric properties, the Professional Care Rating Scale based on Swanson’s Theory (103). This scale has 14 items, seven of which assess the characteristics of a “compassionate healer,” with the authors reporting a Cronbach Alpha of 0.89 among a sample of pregnant women in Colombia (103).

While we had anticipated utilizing the EMPRO tool (Evaluating Measures of Patient-Reported Outcomes), a valid and reliable measure (116) to evaluate the quality of Spanish patient-reported compassion measures (research aim #3) and to determine which measures were best suited for use in a Spanish-speaking patient population (research aim #4), in not identifying any validated Spanish patient-reported measures of compassion we were unable to do so.

## Discussion

The first aim of this study was to determine which tools have been used in Spanish settings to measure compassion. While there has been a rapid influx of research on compassion within Spanish contexts (Figure 2), these are not without limitations. Namely, while the number of publications assessing compassion in Spanish-speaking contexts has increased in recent decades, the number of Spanish studies on this subject is still relatively small compared to data from other countries. While 6.3% of the world population’s primary language is Spanish, making it the world’s second most spoken language by native speakers after Mandarin (117), a recent scoping review on compassion in healthcare revealed that of the 50 studies included in the review, only 4 (8%) of the studies where carried out in Spanish speaking countries, with almost 46 (92%) of the studies being conducted in English speaking countries (4).

Regarding the studies on measuring compassion in Spanish populations reviewed herein, only 3% of studies assessed compassion from the recipient’s perspective, with no studies assessing compassion from the patients’ perspectives specifically (Table 2). In fact, 48% of studies focused exclusively on self-compassion, and 37% focused on compassion for others, all of which were based on responder/provider self-report. This is particularly problematic considering that: (1) the relationship and association between compassion and self-compassion has not been adequately demonstrated (27); (2) Responder/Provider self-reports are biased and do not equate with recipients’ actual experiences of compassion (6, 23) and; (3) compassion is an inherently relational construct (4, 6, 28) which etymologically means “to suffer with another” (118). Despite the necessity of assessing compassion from the recipients’ perspective, only 9% of reviewed studies assessed compassion from others, none of which were translated and validated in Spanish nor included patient populations (see Table 2; Figure 2).

As evident in Table 2, the most suitable instrument for measuring self-compassion in Spanish is the Self-Compassion Scale (65), validated by García-Campayo et al. (34). While there were various instruments measuring compassion toward others in Spanish populations, the Jefferson Scale of Physicians Empathy (JPSE) (74) has been validated in Spanish (66, 67) and is currently the most suitable tool available. However, there are some significant limitations that need to be noted: (1) it is a provider self-report and not a patient-reported measure, and importantly, (2) it was designed and validated to measure empathy which, while sharing attributes of compassion, does not require a pro-social response or action aimed at the alleviation of suffering (27, 28). Researchers have nonetheless argued that two of the 20 items within the scale are relevant to compassion—“taking perspective” and the “ability to put oneself in the patient’s shoes.” Of the two studies that measured compassion with instruments that simultaneously assessed compassion toward others and toward oneself, one used a projective technique and a qualitative approach (112). The other (111) focuses on positive/negative feelings toward self/others and not on the construct of compassion specifically. Neither of these two assessment tools has been validated in Spanish.

In addition, two studies measured compassion from multiple perspectives, namely--from/to others, between others, and to oneself. One focused on assessing the connotative meaning of concepts, and the second measured compassion using the Relational Compassion Scale (RCS) (115). The RCS was designed to measure compassion as received from others; the study that used it assessed compassion from multiple perspectives but did not focus on any one subgroup specifically. Despite these limitations, when considering the assessment tools that could be potentially used to assess compassion in health care settings and from a patient perspective, this tool could be considered after additional validity and reliability testing.

An unexpected and surprising finding was that only six studies measured compassion from the recipients’ point of view, three of which used a measurement tool that had been validated in a Spanish setting, with no study using a patient-reported experience measure (assessment was done with subscales or some items from a broader scale assessing other variables as well). This inhibited our ability to address two of the four research questions related to this study, namely, the research aims: (3) evaluate the quality of these patient-reported measures in Spanish contexts, and (4) determine which of these instruments would be best suited to be used in healthcare settings. The absence of a patient-reported, valid, and reliable, Spanish compassion measure remains a persistent and significant gap in studying, measuring, intervening, and ultimately improving compassion among Spanish-speaking patient populations.

It should also be noted that all the studies that measured compassion received from others used a subset of items from a larger scale. In other words, these studies aimed to evaluate a broader construct that included a facet of compassion that was reputedly linked to compassion received by others. For example, two of the six studies used the Professional Care Rating Scale (115), and one study used the Dental Home Concept Scale (102), which, while being validated in Spanish populations were not designed or validated tools intended for measuring compassion to others specifically.

In addition, the three studies that used a validated tool to assess compassion from others among a Spanish-speaking population were all conducted in the United States and none among patient populations. It is, therefore, likely that the participants were, at least to some extent, familiarized enough with the English language to have an understanding of the meaning of the construct of “compassion” in English, which may or may not be different from conceptualization of compassion in countries were Spanish is the primary language and culture group. This possibility is relevant and needs to be considered because the word “compassion” in Spanish settings may have different connotations than the word “compassion” in English settings (119, 120). In Spanish-speaking cultures, the word “compassion” is often associated with religion and is very closely related to the concepts of mercy or pity (119). In fact, due to this understanding, some studies have operationalized the term for compassion in Spanish as “advantageous inequality” (81), which is not the case in the English-speaking context, where compassion is differentiated from, and preferred over, similar related concepts of empathy and sympathy (27, 28).

As we have already pointed out, the poor results found in this review limited the scope of the third objective of this study. Of the six studies that assessed compassion received from others, only one indicated the instrument’s psychometric properties, the Professional Caregiving Rating Scale based on Swanson’s Theory (103). According to that study, the scale had a Cronbach’s Alpha of 0.89 within the study sample. Even though this is an acceptable reliability score, further studies are needed to consider whether this scale is a valid tool to assess compassion in a healthcare setting from a patient’s perspective. Likewise, in terms of the last aim of this study, the lack of patient-reported compassion measures did not allow us to appraise the most suitable tool to be utilized among Spanish-speaking patient populations. The few studies that aimed to assess compassion received from others were significantly limited as they did so using items from broader assessment tools that were not designed to measure compassion, of which only half of them were validated in Spanish. Considering that compassion is a key element in the care provided in the healthcare context, a patient’s right, an increasingly recognized indicator of quality care, and a critical factor in promoting the patient’s well-being, it is remarkable that there are no valid and reliable instruments to measure compassion in Spanish-speaking patient populations.

Although the authors attempted to carry out a systematic review in accordance with strict methodological guidelines, this review is not free of limitations. As noted, when it comes to measuring compassion in Spanish, researchers have been using instruments that more correctly measure related constructs or sub-components of compassion. This may, in part, be of a larger issue, namely differences between conceptualizations of compassion in Spanish-speaking contexts compared to English-speaking contexts where most measures originally were developed. As a result, one of the limitations of this review is that studies may have been excluded that measured facets of compassion that did not use the term compassion. This underscores the importance of establishing face and construct validity among Spanish populations to determine what constitutes compassion within this context. While this review was restricted to studies that expressly referred to the concept of compassion to ensure methodological rigor, it did include studies that reported measuring compassion, even if it was as a subscale or facet of the broader construct. For example, questionnaires that more correctly measured empathy were included, provided that both the authors of the questionnaire and the researchers who utilized it reported that it measured an aspect of compassion. This was the case with the Jefferson Scale of Physicians Empathy (JSPE), which is the most widely used instrument in Spanish populations aiming to measure compassion toward others.

Despite the growing number of publications in which compassion appears as a variable in Spanish-speaking contexts and the increased interest in the topic among Spanish researchers, there have been no systematic reviews to synthesize and evaluate the evidence associated with this burgeoning field of scholarship. Assessing the experience of compassion in Spanish patient populations could provide a foundation for exploring the importance of compassion in sectors beyond healthcare, such as organizational compassion and within society in general (121).

## Conclusion

While there are valid and reliable measures, validated in Spanish, that measure aspects of compassion, there are no valid and reliable instruments, developed and validated in Spanish, that measure compassion in a comprehensive, methodologically rigorous manner from patients’ perspective. In light of the centrality of compassion to healthcare, quality care ratings, and patient, family, and healthcare staff well-being, there is an increasing urgency to address this gap in order to conduct research and improve care in this area.

## Relevance to clinical practice

In order to ensure and promote compassion in the health care context, it is essential to have a valid and reliable tool to measure this construct in a patient-informed way, and this is currently not possible in the Spanish-speaking context because of the lack of such an instrument in Spanish.

## Data availability statement

The original contributions presented in the study are included in the article/supplementary material, further inquiries can be directed to the corresponding author.

## Author contributions

AS-R: Writing – review & editing, Writing – original draft, Investigation, Formal analysis, Conceptualization. CP: Writing – review & editing. BG-J: Writing – review & editing. YA-V: Supervision, Writing – review & editing, Writing – original draft, Investigation, Funding acquisition, Formal analysis. MP-M: Writing – review & editing. SS: Conceptualization, Writing – review & editing, Supervision, Investigation.
